# Tobacco Use among Emergency Department Patients

**DOI:** 10.3390/ijerph8010253

**Published:** 2011-01-21

**Authors:** Patricia M. Smith

**Affiliations:** Division of Human Sciences, Northern Ontario School of Medicine, 955 Oliver Road, MS2008, Thunder Bay, Ontario, P7B 5E1, Canada; E-Mail: patricia.smith@normed.ca; Tel.: +1-807-766-7341; Fax: +1-807-766-7362

**Keywords:** tobacco prevalence, epidemiology, population surveillance, ED tobacco prevalence, hospital tobacco prevalence, tobacco use identification and documentation

## Abstract

This is the first study to systematically track the tobacco use prevalence in an entire emergency department (ED) population and compare age-stratified rates to the general population using national, provincial, and regional comparisons. A tobacco use question was integrated into the ED electronic registration process from 2007 to 2010 in 11 northern hospitals (10 rural, 1 urban). Results showed that tobacco use documentation (85–89%) and tobacco use (26–27%) were consistent across years with the only discrepancy being higher tobacco prevalence in 2007 (32%) due to higher rates at the urban hospital. Age-stratified outcomes showed that tobacco use remained high up to 50 years old (36%); rates began to decrease for patients in their 50’s (26%) and 60’s (16%), and decreased substantially after age 70 (5%). The age-stratified ED tobacco rates were almost double those of the general population nationally and provincially for all but the oldest age groups but were virtually identical to regional rates. The tobacco use tracking and age-stratified general population comparisons in this study improves on previous attempts to document prevalence in the ED population, and at a more local level, provides a “big picture” overview that highlights the magnitude of the tobacco-use problem in these communities.

## 1. Introduction

Little is known about tobacco use prevalence rates among emergency department (ED) patients, yet this is an important population to understand from a public health perspective and to target with tobacco cessation interventions [1,2]. The few studies that have reported ED tobacco prevalence show great variability (26%–41%) and rates tend to be much higher than the general population [3–6] but firm conclusions have been precluded primarily by two methodological limitations. First, none of the studies have systematically tracked tobacco use on all patients. Data have been based on small samples and surveys with recruitment eligibility criteria representing only a subset of the ED patient population *versus* the entire ED population, thereby introducing sample error. Second, none of the studies have taken age into account. Tobacco use decreases with age, especially after 55+ years [7] so the smoking prevalence for a given population will be directly affected by the proportion of younger to older people included in the sample [8]. If patients <55 years of age represent a relatively larger proportion of the ED population, the tobacco use rates would be expected to be higher than the general population. Even when tobacco use status is electronically recorded as part of standard practice in the ED, the quality of tobacco use data can be poor and data extraction can be difficult due to the inconsistency of asking and recording status by different healthcare providers, lack of standardization in terms of how tobacco use is coded and where it appears in the chart, and various errors such as tobacco status occurring in multiple places in a patient’s chart with discrepant information for the same visit [9].

The current study was designed to move the field forward by addressing methodological issues that have previously hindered conclusions about tobacco use rates in the ED population and by comparing age-stratified prevalence rates with the general population. A standardized tobacco use question that has been used in previous inpatient tobacco cessation studies [8] was chosen and integrated into the ED electronic registration software and ED clerks’ workflow so that the tobacco-use status of the entire ED population could be systematically tracked using the same metric in the same place in the chart. To enhance the specificity of tobacco use prevalence data for public health application, the data were stratified by age and compared to national, provincial, and regional tobacco use prevalence rates for the general population. The long-term perspective of the study was to provide an ongoing assessment of the magnitude of the problem of tobacco use in the participating hospitals and to help inform decision-making about providing tobacco cessation services to patients.

## 2. Experimental Section

### 2.1. Setting

Participating sites were acute care hospitals in the northwest (NW) region of the province of Ontario, Canada. For funding purposes under the provincial single-tier universal healthcare system, the region is defined by the geographical healthcare regionalization structure of the Local Health Integration Network (LHIN). The participating sites represented all 11 of 12 hospitals in LHIN 14 that used Meditech electronic admitting software. The geographical catchment of these hospitals covers a landmass of 526,355 square kilometers, almost 60% of the province, and includes over 30 northern and remote First Nations communities, but it represents only 2% of the provincial population (approximately 235,000 of which 20% are Aboriginal peoples) with a population density of 0.5 persons/km^2^ compared to 12.5 persons/km^2^ provincially [10]. One of the hospitals was the regional tertiary hospital located in an urban centre (Thunder Bay); the other 10 hospitals were in the rural communities of Atikokan, Dryden, Fort Frances, Geraldton, Marathon, Manitouwadge, Nipigon, Red Lake, Sioux Lookout, and Terrace Bay. All 11 hospitals have recently become affiliated with a new medical school as northern, rural, and remote teaching sites for undergraduate and graduate learners. The study received ethics clearance through the regional hospital institutional review board (IRB), which also served as the IRB for 8 of the community hospitals; 2 of the community hospitals provided individualized clearance from their own IRBs.

### 2.2. Measures

Tobacco use status was defined as 30-day point prevalence: “Have you smoked or used any tobacco products in the last 30 days?” (yes, no, not asked, not able [to answer], and refused). Thirty days allows comparison to inpatient studies [11,12]. Although the question was phrased as “smoked or used any tobacco products”, previous studies have showed that 97–99% of hospital patients who report using tobacco smoke cigarettes [12]. Demographic data that were part of the regular ED registration process were extracted for analyses to characterize the population—gender, age, hospital site, and date. To protect confidentiality, patient age instead of the more specific birth date was extracted and names were not recorded. Hospital-assigned medical record numbers were extracted to enable statistical control for repeat visits; the researchers had no way to track these numbers to patient names or other personally-identifying information.

Statistics Canada and Health Canada cross-sectional survey data were used for comparative tobacco use prevalence data for the general population—community level prevalence data were not available but national, provincial, and regional data were. The regional data were available by public health district; there are two public health districts in NW Ontario—Northwestern District Public Health (NWDPH) and Thunder Bay District Public Health (TBDPH). The most recent and complete population data were used for population comparisons—Health Canada’s 2007 Canadian Tobacco Use Monitoring Survey for national and provincial rates [7], and Statistics Canada 2009 socioeconomic database CANSIM for regional rates [13]. The general population data are specific to smoking prevalence, not all tobacco use.

### 2.3. Procedure

The Chief Executive Officer and senior management team in each hospital in LHIN 14 were approached with the opportunity to participate in the research project. A presentation was made that included: (1) what adding a tobacco use question to the admitting records would involve; (2) clinical practice guideline recommendations for institutionalizing tobacco use identification and documentation [2]; (3) the type of data and reports that would be available to the hospitals resulting from the systematic tracking of tobacco use; and, (4) accreditation benefits to the hospitals for research participation and disease surveillance activities. Hospitals agreed to add the tobacco use question on the admitting records to be asked by admitting staff and agreed to allow the data to be extracted and analyzed for research purposes. They also arranged for the admitting staff to be trained by the principal investigator (PI).

The researchers worked with the regional hospital’s information services (IS) department to add the question to the electronic admitting records of all participating hospitals and to “turn on” the question at each hospital only after the admitting staff had been trained. For quality control, the question was added as a no-by-pass field, and reasons for incomplete data were added as options for the question (not asked, not able [to answer], and refused) so they could be tracked. The Meditech admitting databases for all of the participating hospitals were connected to the regional hospital’s system through a shared licensing agreement which simplified and centralized the process. The admitting staff received brief training from the PI that covered their role in asking the tobacco-use question and details about the question—where to find it in Meditech, how to ask and answer the question, the fact that the system had been set so that the question could not be by-passed, how to answer patients’ questions about being asked the question, and how the responses from the question would be used to determine tobacco use prevalence so that in turn, the magnitude of the need for providing patient services for tobacco cessation could be determined. Posters were placed in the admitting areas that explained the study.

All patients visiting the ED in the participating hospitals were registered by admitting clerks as part of standard practice. Admitting clerks were instructed to ask all patients 18+ years about their tobacco use status. Scheduled visits such as dressing changes and IV anti-biotic administration were not eligible nor were appointments for laboratory work or other technical services that were not emergency visits. Data were extracted from all hospitals’ Meditech database by a program written by the regional hospital’s IS department, and made available to the researchers through a SQL server purchased for the study.

### 2.4. Primary Outcomes

The primary outcomes were proportion of patients screened for tobacco use, overall 30-day point prevalence tobacco use, tobacco prevalence stratified by age category, and age-stratified tobacco prevalence for ED patients compared to regional, provincial, and national prevalence in the general population.

### 2.5. Statistical Analyses

Data were cleaned to exclude patients <18 years of age and non-emergent visits as per the eligibility criteria (e.g., dressing changes, IV medications, lab visits, *etc.*). Tobacco prevalence was calculated separately for each year of data collection (January–December 2007–2010) using frequency counts. Each individual patient was counted only once per year using the first admission for the year; all other visits were excluded to maintain independence of data. Age categories for the main tobacco use prevalence outcomes were based on the 5-year age categories used by Statistics Canada for population census data [10]. Larger age categories were used for comparisons with the general population based on age categories for which regional, provincial, and national smoking prevalence data were available. Patients 18 and 19 years of age were excluded from the provincial and regional population comparisons because general population smoking prevalence data combine 18–19 year olds with younger ages (12–15 years) and the current study did not collect data on patients <18 years. For the regional comparisons, the communities of Atikokan, Fort Frances, Red Lake, Sioux Lookout, and Dryden were included in the Northwestern Public Health District, and Thunder Bay, Nipigon, Geraldton, Terrace Bay, Marathon, and Manitouwadge were included in the Thunder Bay Public Health District.

## 3. Results and Discussion

The screening and tobacco use outcomes are presented in Table 1. The total number of ED visits averaged 136,338/year. When each patient’s first visit each year was used to censor the data, approximately 50% of records were excluded for the tobacco use prevalence analyses for each year of the study (Table 1). Tobacco screening rates were consistent across all four years (85–89%) and between the community hospitals and the regional hospital. Among patients screened for tobacco use, 48% were male, the average age was 46 ± 19, and 69% of all visits were accounted for by patients less than 55 years of age.

Overall tobacco use remained relatively stable across the years (26–27%) with the only discrepancy being higher rates in 2007 (32%; Table 1). Although the overall tobacco use rates are high compared to the 18% provincial and national averages [7], they are consistent with overall regional averages for the general population which are the highest in the province [13], likely due, at least in part, to a large blue collar population (mining, logging, and pulp/paper mills), lower socio-economic status compared to provincial averages, and a large First Nations population [10] all of which are associated with higher smoking rates [14–16].

**Figure 1:**
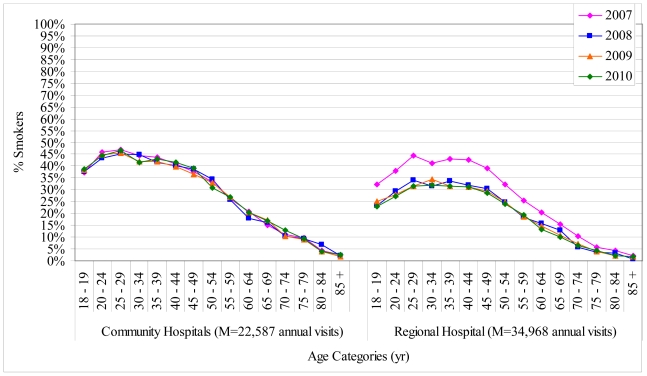
Tobacco Use Prevalence for Community and Regional Hospitals by Age and Year (2007**–**2010).

Showing tobacco use prevalence in the 5-year age categories chosen for this study helped to define exactly where tobacco use prevalence decreased by age (Table 1 and Figure 1). These data are unique—although population data show that prevalence rates decrease with age, most published tables have an age ceiling of 45+, 55+, or 65+ and use larger groupings than 5-years and thus do not clearly show where the threshold for change in prevalence lies. The current findings show that tobacco use prevalence remained over 30% for each five-year age group until age 50 (36% overall for patients <50 years). Prevalence decreased to 26% for patients in their 50’s, decreased to 16% for patients in their 60’s, and decreased substantially to 5% for patients 70+ years.

Follow-up analyses showed that the decrease in tobacco prevalence from 2007 to 2008 was due to a 1% decrease in the community hospitals (owing to a drop from 43% to 42% among patients <50 years) and an 8% decrease in the regional hospital, which was most pronounced for patients <70 years of age—10% decrease among patients <50 years (41% to 31%), 6% decrease for 50–69 years (25% to 19%), and 2% decrease for 70+ years (6% to 4%; Figure 1). There is no clear explanation for the dramatic drop in prevalence at the regional hospital from 2007 to 2008—there were no new community-level smoking cessation initiatives, municipal smoking bylaw changes, or tobacco cost changes that were different in the urban centre *versus* the rural communities, and no identifiable changes at the regional hospital that could have been responsible for a change in patient population such as fewer visits from people living outside of Thunder Bay in the rural communities. Although it is possible that the 2007 decrease was a systems error at the regional hospital, it is unlikely because the overall number of ED visits, the proportion of visits by age group, the proportion of patients asked about tobacco status, and the overall pattern of tobacco use by age group remained the same from 2007–2008.

The follow-up analyses to explore the decrease in prevalence from 2007–2008 revealed that the average prevalence rate for the community hospitals (33%) was 8% higher than the regional hospital (25%)—3% higher for 2007 (34% *vs.* 31%) and an average 10% higher for the other years—33% *vs.* 23% for 2008, 32% *vs.* 23% for 2009, and 32% *vs.* 22% for 2010. For 2008–2010, the most notable difference was for patients under 30 years of age for whom prevalence was 15% higher for rural *versus* urban communities; for the other age groups, the rural hospital tobacco use rates compared to the regional hospital were 9% higher among patients 30–49 years, 7% higher among patients 50–69 years, and 3% higher among patients 70+ years. The 8% overall higher rate for the rural community hospitals across all four years is consistent with a 7% rural-urban difference in smoking prevalence in a recent report on the health status of rural Canadians [17]. The general population prevalence rates in the report were also virtually identical to the current findings—32% for rural communities and 25% for urban centers [17]. The age-stratified findings from the current study contribute new data to rural-urban differences.

**Figure 2:**
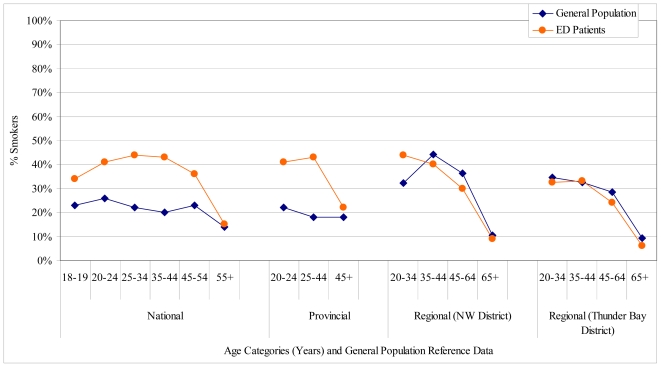
Comparison of Tobacco Use Prevalence between the ED Population and the National, Provincial, and Regional General Populations. Note: The general population national and provincial data are from Health Canada, 2007 [7]; the regional data are fromStatistics Canada, 2009 [13]; the corresponding ED data are from 2007 (Table 1) for national and provincialcomparisons and 2009 for regional comparisons.

Comparisons between the tobacco use prevalence of the ED patients and the general population are in Figure 2. The ED tobacco use prevalence was basically double the national and provincial averages up to age 44, almost 60% higher for 45–54 year olds nationally, and very similar to both the provincial and national averages for the oldest age groups (55+ nationally and 45+ provincially). Despite these elevated rates compared to national and provincial averages, the ED tobacco use rates were similar to age-stratified regional rates with a few exceptions—prevalence for ED patients 20–34 years in the NW Health Unit District was 12% (absolute) higher than the general population and prevalence for ED patients age 45–64 were 5–6% lower than the general population in both districts.

## 4. Conclusions

This is the first study to systematically track the tobacco use prevalence in an entire ED population, present the prevalence stratified by age, and compare age-stratified rates to the general population using national, provincial, and regional comparisons. The findings revealed that despite an overall national downward trend in smoking nationally, from 50% in 1960 to 26% in 1999 [18] to 18% in 2008 [19], the rates in the participating communities remain high, at 1999 national rates. From a methodological perspective, the results show how important it is to consider age and rural-urban differences, and to make population comparisons that include data from the communities or regions being studied. Tobacco prevalence was high overall and across all but the oldest age groups, but “high” was relative to provincial and national comparisons because regionally, the rates were consistent with smoking rates in the general population. From an epidemiological perspective, it is not clear why the tobacco use rates for the oldest patients were the same as the national and provincial averages when the rates for younger patients were double that of national and provincial averages. There is no evidence that smokers in this region are quitting at a rate any faster than the national and provincial quit rates to explain the sudden drop to national averages for older patients; in fact, the prevalence rates in NW Ontario remain elevated, similar to the 1999 national rates [18], suggesting that people are not quitting as quickly. It is possible that smokers after age 55 are either moving away from these northern communities permanently or seasonally. Since tobacco is the primary preventable cause of premature death [20], it is also possible that some of the smokers are dying prematurely.

Adding the tobacco use question to the ED registration as an electronic non-clinician-based system centralized the process of identifying and documenting tobacco use consistent with clinical practice guidelines [2], allowed standardization of the wording and location of tobacco use in the chart thereby contributing to the ease of data extraction and analysis, and resulted in the ability to systematically track the status of the entire ED population rather than only a select sample. The centralized approach also confined training to a small cadre of registration clerks and allowed a narrow focus on asking and documenting tobacco use rather than having to train a large number of clinicians who would have required a broader focus on asking about tobacco use and following through with the other components of the 5A brief intervention protocol (ask, advise, assess, assist, arrange) recommended by the Public Health Service (PHS) guideline [2]. The centralization also eliminated the need to rely on the workloads and willingness of a multitude of clinicians to ask and document tobacco use consistent with the PHS guideline. Although it was not done for this study, the centralized tobacco use question can be used to trigger cessation interventions by clinicians.

There were benefits to the hospitals beyond the research itself. Institutionalizing tobacco use identification and documentation procedures is recommended by clinical practice guidelines [2] and through participation in this study, hospitals were able to implement this best practice. Hospitals were also able to use participation in research and implementation of a best practice in their hospital accreditation reports as a quality indicator for disease surveillance/prevention although tobacco-use identification and documentation is not a specific, nor mandatory, accreditation standard in Canada.

There were limitations to this study. First, tobacco use status was self-reported by patients, it was not recorded for 11% of admissions each year, and the “twelfth” hospital in NW Ontario did not contribute data because they were not on the same Meditech electronic system as the other hospitals thereby limiting the generalizability to the entire region. However, the number of patients tracked was sufficiently large and tobacco use status was tracked over a long enough period of time that the data collected can reasonably be considered to represent the ED population for this northern region of the province rather than a sample of the population. Second, although the ED tobacco use prevalence data were similar to that of the general population of the region and remained stable over time, people who visit the ED could be different than the general population so the generalizability of the ED prevalence rates to the general population should be interpreted with caution. Finally, due to our inability to explain the dramatic drop in tobacco prevalence from 2007 to 2008 at the regional hospital, the higher prevalence rates for 2007 at the regional hospital should be interpreted with caution.

The systematic tobacco use tracking and general population comparisons in this study provide a “big picture” overview that highlights the magnitude of the tobacco use problem in these northern communities and will enable those responsible for tobacco control policies, programs, research, and surveillance to assess the situation and inform decision-making. In order to allocate limited healthcare resources rationally for tobacco control, it is necessary to elucidate the economic burden of tobacco use within different segments of the population. The age-specific tobacco use prevalence in these communities is now well described for variability and the tobacco use question remains on the ED registration software as part of standard practice so tobacco use can continue to be collected and used to track trends over time. The ability to systematically track tobacco use on the ED population and the findings that revealed similar tobacco use prevalence rates between the ED and general population also highlight the possibility of using the ED for other surveillance purposes, especially in smaller communities where community-specific population data might not be available.

## Figures and Tables

**Table 1:** Tobacco Use Screening and Prevalence by Year.

	2007		2008		2009		2010	
	N	%	N	%	N	%	N	%
Total visits	120,927	100%	132,211	100%	147,286	100%	144,928	100%
Individuals	60,273	50%	65,450	50%	67,920	46%	68,461	47%
Screened	53,895	89%	57,729	88%	59,763	88%	58,830	85%
Refused	60	<1%	78	<1%	15	<1%	27	<1%
Not able	1,466	2%	1,332	2%	1,165	2%	1,222	2%
Not asked	4,440	7%	6,217	10%	6,152	9%	7,471	11%
Blank	412	1%	94	<1%	825	1%	911	1%
Males	25,962	48%	27,680	48%	28,399	48%	28,136	48%
Age *M, *±*SD, range*	46 ± 19 (18–107)		46 ± 19 (18–103)		46 ± 19 (18–105)		47± 19 (18–106)	
Smokers by age								
18–19 yr	885/2,595	34%	835/2,878	29%	887/2,954	28%	791/2,733	29%
20–24 yr	2,423/5,973	41%	2,108/6,098	35%	2,169/6,452	34%	2,076/6,203	34%
25–29 yr	2,211/4,868	45%	1,983/5,142	39%	1,983/5,334	38%	1,948/5,159	38%
30–34 yr	1,880/4,410	43%	1,718/4,611	37%	1,788/4,793	36%	1,676/4,642	36%
35–39 yr	1,962/4,515	44%	1,776/4,811	37%	1,731/4,827	37%	1,685/4,622	36%
40–44 yr	2,040/4,878	42%	1,715/4,849	35%	1,694/4,873	35%	1,634/4,608	36%
45–49 yr	1,981/5,143	38%	1,851/5,442	34%	1,787/5,522	34%	1,721/5,192	33%
50–54 yr	1,496/4,566	33%	1,445/5,004	29%	1,497/5,333	27%	1,441/5,349	27%
55–59 yr	1,001/3,888	26%	926/4,332	21%	988/4,497	23%	1,023/4,521	23%
60–64 yr	636/3,110	20%	578/3,483	17%	649/3,805	16%	650/4,001	16%
65–69 yr	375/2,438	15%	387/2,742	14%	369/2,784	13%	369/2,876	13%
70–74 yr	229/2,167	11%	183/2,365	8%	206/2,468	9%	236/2,637	9%
75–79 yr	142/2,090	7%	131/2,268	6%	136/2,298	6%	142/2,300	6%
80–84 yr	75/1,736	4%	86/1,960	4%	57/1,992	3%	56/1,982	3%
85+ yr	34/1,518	2%	22/1,744	1%	33/1,831	2%	43/2,005	2%
Total	17,370/53,895	32%	15,744/57,729	27%	15,974/59,763	26%	15,491/58,830	26%
